# An Affirmative Coping Skills Intervention to Improve the Mental and Sexual Health of Sexual and Gender Minority Youth (Project Youth AFFIRM): Protocol for an Implementation Study

**DOI:** 10.2196/13462

**Published:** 2019-06-06

**Authors:** Shelley L Craig, Lauren B McInroy, Andrew David Eaton, Gio Iacono, Vivian WY Leung, Ashley Austin, Cheryl Dobinson

**Affiliations:** 1 Factor-Inwentash Faculty of Social Work University of Toronto Toronto, ON Canada; 2 College of Social Work The Ohio State University Columbus, OH United States; 3 Ellen Whiteside McDonnell School of Social Work Barry University Miami Shores, FL United States; 4 Planned Parenthood Toronto Toronto, ON Canada

**Keywords:** sexual and gender minorities, youth, coping behavior, pragmatic clinical trial, cognitive behavioral therapy, implementation science

## Abstract

**Background:**

Sexual and gender minority youth (SGMY, aged 14-29 years) face increased risks to their well-being, including rejection by family, exclusion from society, depression, substance use, elevated suicidality, and harassment, when compared with their cisgender, heterosexual peers. These perils and a lack of targeted programs for SGMY exacerbate their risk for HIV and other sexually transmitted infections. Cognitive behavioral therapy (CBT) interventions support clients by generating alternative ways of interpreting their problems and beliefs about themselves. CBT, tailored to the experiences of SGMY, may help SGMY improve their mood and coping skills by teaching them how to identify, challenge, and change maladaptive thoughts, beliefs, and behaviors. Based on the promising results of a pilot study, a CBT-informed group intervention, AFFIRM, is being tested in a pragmatic trial to assess its implementation potential.

**Objective:**

The aim of this study is to scale-up implementation and delivery of AFFIRM, an 8-session manualized group coping skills intervention focused on reducing sexual risk behaviors and psychosocial distress among SGMY. Our secondary aim is to decrease sexual risk taking, poor mental health, and internalized homophobia and to increase levels of sexual self-efficacy and proactive coping among SGMY.

**Methods:**

SGMY are recruited via flyers at community agencies and organizations, as well as through Web-based advertising. Potential participants are assessed for suitability for the group intervention via Web-based screening and are allocated in a 2:1 fashion to the AFFIRM intervention or a wait-listed control in a stepped wedge wait-list crossover design. The intervention groups are hosted by collaborating community agency sites (CCASs; eg, community health centers and family health teams) across Ontario, Canada. Participants are assessed at prewait (if applicable), preintervention, postintervention, 6-month follow-up, and 12-month follow-up for sexual health self-efficacy and capacity, mental health indicators, internalized homophobia, stress appraisal, proactive and active coping, and hope. Web-based data collection occurs either independently or at CCASs using tablets. Participants in crisis are assessed using an established distress protocol.

**Results:**

Data collection is ongoing; the target sample is 300 participants. It is anticipated that data analyses will use effect size estimates, paired sample *t* tests, and repeated measures linear mixed modeling in SPSS to test for differences pre- and postintervention. Descriptive analyses will summarize data and profile all variables, including internal consistency estimates. Distributional assumptions and univariate and multivariate normality of variables will be assessed.

**Conclusions:**

AFFIRM is a potentially scalable intervention. Many existing community programs provide safe spaces for SGMY but do not provide skills-based training to deal with the increasingly complex lives of youth. This pragmatic trial could make a significant contribution to the field of intervention research by simultaneously moving AFFIRM into practice and evaluating its impact.

**International Registered Report Identifier (IRRID):**

DERR1-10.2196/13462

## Introduction

### Intersecting Vulnerabilities of Sexual and Gender Minority Youth

Compared with their cisgender, heterosexual peers [1-8], sexual and gender minority youth (SGMY, aged 14-29 years) face increased risks to their well-being, including rejection by family [9], exclusion from society [10], depression [10,11], substance use [12], elevated suicidality [13,14], and harassment [15-19]. In the pilot study that informed this protocol, participants attributed the stress of their SGM status as a significant contributor to their risks [20]. Yet, there remains a dearth of research and services for SGMY [21]. Existent risks and the lack of targeted programs [21-25] exacerbate their risk for HIV infection [12,26]. In 2013, nearly 25% of the 2090 Canadians diagnosed with HIV were aged between 15 and 29 years, and 43% of those youth contracted HIV through same-sex exposure (compared with only 26% in 2004) [12,26,27].

There is a particular lack of research with female-identified SGMY, who report higher rates of HIV-related risk behaviors than their male-SGMY and non-SGMY peers [28-37]. These risks include sex with multiple partners [28,29], unprotected vaginal intercourse [30], injection drug use [31], and pregnancy [32,33]. SGMY risk factors are further increased in female-identified SGMY, as they have higher rates of mental health concerns compared with male-identified SGMY, including depressive symptoms and suicidality at younger ages, suggesting earlier onset of co-occurring concerns [34-36].

Thus, multiple factors such as depression [10], sexual health-risk behaviors [31], discrimination [16] and perceptions that HIV is not a threat [28-30] may interact to exacerbate SGMYs’ risk [3]. Female-identified SGMY—as well as transgender, gender diverse, and racialized SGMY—experience even greater vulnerabilities to mental health stressors, which in turn can exacerbate sexual health risk [38-40]. Holistic interventions that affirm SGMY identities and cultivate a sense of community, may mitigate these risks [41,42]. As nearly 70% of premature adult deaths are related to behaviors initiated in adolescence (eg, unsafe sex and substance abuse) [43], this age range is a critical period to implement interventions that help youth cope with the risks they experience.

### Theoretical Approach

Syndemics, minority stress, and community-based research theories provide insight toward SGMY intervention development. Syndemics theory highlights social inequities as root causes of synergistic interaction of 2 or more coexistent issues (eg, SGMY status and depression) or mutually reinforcing epidemics (eg, HIV) contributing to health disparities among marginalized populations [40,44-47]. Vulnerable individuals may encounter lifelong adversity, particularly from social marginalization and stigma [45], posing a greater risk for problems [44,46] that can lead to poorer sexual and health outcomes [47]. For SGMY, as the number of psychosocial health problems increases, the risk of major negative health outcomes also increases [47-49]. To comprehensively combat sexual health risks, overlapping vulnerabilities must be addressed concurrently [48,49].

Minority stress theory posits that marginalized populations experience a unique form of stress because of conflict between their identities and social expectations [50-53]. It partially explains why SGMY encounter disproportionate chronic stress, discrimination, and victimization [52], which subsequently increases likelihood of sexual risk taking, depressive anxiety, and substance abuse disorders [52,53]. An influential stressor, internalized homophobia (negative beliefs about one’s own SGMY status) [54], is related to unprotected sex [54,55] and increased depression [10]. Minority stress may increase internalized homophobia and stress-related cortisol production associated with heightened depression, anxiety, and suicidal ideation [50,54]. SGMY may not learn to cope with stressors through traditional means (eg, family or community support) [55-58], resulting in vulnerability to health and mental health threats [48] and increasing likelihood of engaging in risky behaviors [49]. As traditional approaches do not address many of the co-occurring stressors for SGMY, interventions that enhance coping skills are critical [59-82].

Building on a rich history of community engagement in HIV/AIDS service delivery, community-based research is critical to intervention development with SGMY, particularly in diverse communities [83-88]. Community-based approaches build on shared values, belief systems, and social practices, allowing for discussions of HIV and sexual health-risk behaviors in a culturally sensitive manner [84-87]. Youth interventions developed in partnership with community also have a much higher rate of agency adoption than those with only academic stakeholders [88]. It is increasingly recognized that future youth interventions must include nimble design and flexible delivery [89-91]. Community feasibility studies improve an intervention’s implementation potential while maintaining rigor in evaluation [92,93].

### Affirmative Cognitive Behavioral Therapy

Cognitive behavioral therapy (CBT) suggests that people’s behaviors and emotions are influenced by their perceptions of life events [60-62] and how one interprets their situation will impact the way they feel or behave [63]. As an example, a person who is depressed may experience unhelpful interpretations or perceptions of themselves because of their problems and life events [61,62]. CBT interventions support clients by generating alternative ways of interpreting their problems and beliefs about themselves [59,63]. Generating alternative ways of thinking and beliefs may facilitate positive changes in one’s behaviors and emotional states [64].

CBT tailored to the experiences of SGMY may help improve mood and increase coping by teaching youth how to identify, challenge and change maladaptive thoughts, beliefs, and behaviors [59,66,67]. Continuing to practice CBT skills after each session (ie, via an action plan) further strengthens adaptive and affirming beliefs and behaviors. This process may lead to changing deeply ingrained problematic ways of thinking and behaving [65-67]. The process of identifying and challenging unhelpful beliefs about sexual and gender identities in an affirming and supportive environment may facilitate a decrease in internalized homo-, bi-, and transphobic cognitions and emotions and lead to improvement in mood and coping [67-69]. Ultimately, CBT within an affirmative therapeutic context can support SGMY in challenging maladaptive coping skills (eg, negative beliefs, isolation, substance misuse, and self-harm) and learning adaptive coping skills (eg, balanced thinking, enhancing social supports, and goal-setting) through education, modeling, practicing skills, and positive reinforcement [15,69]. 

CBT has been effective at treating depression and sexual health-risk behaviors among minority status adolescents [70-72] and lesbian, gay, bisexual, transgender, and queer (LGBTQ) adults [66,72]. However, its effectiveness for SGMY is largely unknown [69]. While the majority of studies do not assess long-term treatment gains, some evidence suggests that such interventions have mental and sexual health benefits for minority populations [79]. Longitudinal research is needed to determine the sustained effectiveness of CBT interventions on adolescent and young adult SGM populations [80]. A few existing studies show promise with sustained reductions in depression found at 12 months [76,77] and 18 months [78], as well as sexual risk of young men who have sex with men at 6 months [72]. Despite the calls for resilience and coping-based research and interventions for SGMY [80], scholarship has maintained a focus on negative health and psychosocial outcomes [81]. To date, no studies have identified the utility of a large-scale implementation of CBT tailored for community-based, diverse groups of SGMY and drawing on an affirmative approach. This study is designed to fill that gap.

## Methods

### AFFIRM Structure

This pragmatic trial is designed to evaluate AFFIRM, a manualized group intervention for SGMY, which follows the structure described in Textbox 1 and is described in more detail elsewhere [20].

**Description of AFFIRM intervention (session focus and session activities).**

Session 1 focus

- Introduction to cognitive behavioral therapy (CBT), exploring lesbian, gay, bisexual, transgender, and queer (LGBTQ)+ identities, and understanding minority stress.

Session 1 activities

- Introductions
- Discussing the theory and purpose of CBT approaches
- Exploring stress and minority stress
- Understanding the causes of stress in our lives

Session 2 focus

- Understanding the impact of anti-LGBTQ attitudes and behaviors on stress.

Session 2 activities

- Check in and review
- Examining homophobia, heterosexism, and transphobia at the individual, institutional, and cultural level
- Identifying how these experiences impact thoughts, feelings, and behaviors
- Fostering strategies for both coping with and combating anti-LGBTQ discrimination at all levels

Session 3 focus

- Understanding how thoughts impact feelings.

Session 3 activities

- Check in and review
- Distinguishing between thoughts and feelings
- Exploring how thoughts influence feelings and behaviors
- Identifying counterproductive thinking patterns
- Recognizing negative self-talk and feelings of hopelessness
- Learning thought stopping

Session 4 focus

- Using thoughts to change feelings.

Session 4 activities

- Check in and review
- Increasing positive thinking and feelings of hope
- Changing negative thoughts to positive thoughts
- Challenging negative thinking and internalized homophobia/negative feelings through the ABCD (activating event, belief, consequence, and debate) method

Session 5 focus

- Exploring how activities impact feelings.

Session 5 activities

- Check in and review
- Examining the impact of various activities on feelings
- Identifying supportive and identity-affirming activities
- The impact of LGBTQ-affirming activities on feelings

Session 6 focus

- Planning to overcome counterproductive thoughts and negative feelings.

Session 6 activities

- Check in and review
- Distinguishing between clear and unclear goals
- Identifying short, mid-, and long-term goals
- Creating a sexual health plan
- Fostering hope for the future

Session 7 focus

- Understanding the impact of minority stress and anti-LGBTQ attitudes or behaviors on social relationships.

Session 7 activities

- Check in and review
- Anti-LGBTQ discrimination can lead to feelings of discomfort around others
- Responding to discrimination or harassment in social situations
- Learning to be assertive

Session 8 focus

- Putting it all together: developing safe, supportive, and identity-affirming social networks.

Session 8 activities

- Check in and review
- Maintaining a healthy social network: attending to thoughts, expectations, feelings, and behaviors within relationships
- Identifying a plan for building a supportive network

### Research Questions, Hypotheses, and Objectives

This project is intended to scale-up implementation and delivery of AFFIRM, an 8-session manualized group coping skills intervention focused on reducing sexual risk behaviors and psychosocial distress among SGMY. This project aims to decrease sexual risk taking, depression, and internalized homophobia and increase levels of sexual self-efficacy and proactive coping among SGMY (ages 14-29 years). This project has the following research questions:

1. To what extent can AFFIRM be feasibly implemented in a range of practice settings, such as community health centers, family health teams, and community-based organizations?
2. What are the facilitative conditions and implementation barriers to effective delivery of AFFIRM?
3. How does participation in an affirmative coping skills intervention (AFFIRM) impact the psychosocial distress and sexual self-efficacy of SGMY?

Given these research questions, the study has the following hypotheses:

Hypothesis 1: AFFIRM can be feasibly implemented in a range of practice settings, and SGMY will have high rates of acceptability of the intervention.

Hypothesis 2: SGMY participating in AFFIRM will show significantly greater decreases in psychosocial distress (eg, internalized stigma and depression) compared with wait-listed controls (ie, SGMY attending existing community programs).

Hypothesis 3: SGMY participating in AFFIRM will show significantly higher levels of sexual self-efficacy and coping compared with wait-listed controls.

### Eligibility Criteria

#### Inclusion and Exclusion Criteria

Inclusion criteria are as follows:

- Aged 14 to 29 years at the time of screening
- Identifies as a sexual and/or gender minority
- Reads, writes, and speaks fluent English
- Is interested in participating in the 8-session AFFIRM Intervention.

Exclusion criteria are as follows:

- Assessed by the Facilitation Team to be in crisis (ie, high risk of suicidality)
- Warranting a more intensive intervention

#### Trial Design

This pragmatic quasi-experimental study uses a stepped wedge wait-list crossover design (SWWCD), whereby all participants receive the intervention in clusters [94-98]. SWWCD has been utilized in community-based research where traditional randomization with a no-treatment condition is unethical, unacceptable to community stakeholders, or not feasible [98]. This study will examine the effects of participating in an AFFIRM intervention group (each consisting of 6-10 participants) compared with wait-list for SGMY (ages 14-29 years).

##### Randomization

Randomization is not possible in this study because of participants’ registration through various CCAS.

##### Blinding

Participants are not blinded as they know whether they are assigned to intervention or wait-listed control. Facilitators are blinded to outcome assessments as outcomes are administered via survey weblink.

##### Allocation

Participants are allocated in an approximate 2:1 fashion to intervention: wait-listed control. This ratio is based on the availability of practice sites to implement AFFIRM in their clinical practices [96,97]. Importantly, similar to community programming, the groups are constructed to be developmentally appropriate. Participants in a particular intervention group are typically within an age range of 3 to 5 years. For example, a 14 year old would generally not be placed in a group with anyone older than 18 years. Groups of people aged between 18 and 29 years may have a broader age range, as developmental stage is not as relevant for established adults. AFFIRM also consists of mixed identity groups (eg, sexual identities and gender identities), based on community feedback. This means that a single intervention group could comprise SGMY from a range of identities. Many community organizations do not focus on 1 particular SGM subpopulation but instead serve all SGMY.

##### Framework

This pragmatic trial is designed to assess AFFIRM’s implementation potential in real-world practice conditions.

#### Study Setting

Collaborating community agency sites (CCASs) in a variety of urban, suburban, and rural communities across Ontario, Canada, are hosting a series of AFFIRM intervention groups, each consisting of 6 to 10 SGMY (aged 14-29 years). At present, there are 23 CCAS, of which 12 are urban, 8 are suburban, and 3 are rural, with more communities likely to be added in subsequent years of the study.

#### Justification

Age group of 14 to 29 years have been identified as a crucial time for SGMY as they start to come out, address family issues, and transition to postsecondary education and early employment [1-3]; as such, this age may be the ideal time for an affirmative CBT intervention. As SGMY face greater well-being risks than their cisgender, heterosexual peers, queerness is also an important qualifier for this study [4-8]. This study focuses on English-speaking CCASs in Ontario. Commitment to an 8-session intervention and exclusion because of crisis are criteria common to group intervention research [99]**.**

### Interventions

#### AFFIRM Intervention (Experimental)

AFFIRM aims to help SGMY develop coping skills through a combination of education (delivered by facilitators) and rehearsal (ie, simulation of real-life experiences) in a manner that affirms (ie, validates) participants’ sexual and gender minority identities, as well as their experiences. Affirmations are explicated through (1) acknowledging and validating the unique struggles experienced by SGMY (eg, homophobia); (2) exploring how participants currently cope with SGM-specific stressors (eg, familial disapproval); (3) facilitating the development of realistic alternative ways of thinking and behaving that affirm youth identities and sexual health choices while integrating healthy ways of coping with internal/external stressors; and (4) enhancing social connection between participants. AFFIRM also includes an overview of sexually transmitted infections (STIs), HIV/AIDS and hepatitis C, and focuses on activities that promote harm reduction, such as a personalized sexual safety plan regardless of accumulated sexual experience. Each series of AFFIRM begins with an orientation session for introductions and discussion of the 8-session schedule. Each of the 8 group sessions of AFFIRM then consist of (1) warm-up/review; (2) discussion of session objectives; (3) behavioral activities; (4) practice and rehearsal; and (5) group reflection and summary.

#### Wait-List (Control)

If they choose, wait-listed participants will attend existing community programs, considered to be treatment as usual for community intervention studies. In a SWWCD study, sites offer iterations of the intervention in phases; individuals in this study will move from the wait-list to AFFIRM over time [94]. The wait-list time frame is minimized as much as possible to ensure an ethical research process. As it is increasingly acknowledged that evidence-based practice requires community and practice-based research [95], alternative intervention designs that are rigorous and work in *the real world* are needed. Such designs adapt to local needs and often have better intervention outcomes [96-100].

#### Discontinuation Criteria

Participants who are in crisis (eg, actively suicidal) at any point during their in-person participation in the AFFIRM intervention are immediately assessed, and appropriate steps are taken to address their individual situation—up to and including being taken to local support services or hospitals by AFFIRM facilitators. Participants who are in crisis are withdrawn from the intervention and the study. A distress protocol (Multimedia Appendix 1) and crisis response form (Multimedia Appendix 2) have been developed for AFFIRM facilitators and is systematically implemented throughout the intervention when participants indicate signs of distress. Importantly, to enhance participants’ safety and well-being, as part of the Web-based data collection, participants are asked at various points if they need immediate help and are provided with immediate resources, including national crises organizations (such as Kids Help Phone Canada or the Trevor Project) with 24-hour counseling via phone, chat, and/or SMS text messaging (short message service, SMS).

#### Protocol Adherence Strategy

The Core Facilitation Team (delivering the AFFIRM intervention) comprises social workers with a master’s degree who are trained in AFFIRM, who meet weekly with the principal investigator to review study progress and ensure protocol adherence. All iterations of AFFIRM implemented in this study are cofacilitated by (1) one of the members of the Core Facilitation Team and (2) 1 representative of the CCAS (eg, a social worker, nurse, or community worker) where the intervention group is being held or another community-based professional, all of whom are trained in the AFFIRM intervention.

#### Concomitant Care and Interventions

There are no restrictions on participant involvement in other studies and/or interventions as a result of their participation in this project. While this prevents accounting for confounding factors (eg, combined effect of participating in another intervention), the geographic context of services in Ontario is such that it is unlikely that participants would have access to SGMY-specific affirmative interventions outside of this study.

### Outcomes

#### Primary

The primary outcomes of this study are feasibility and acceptability. Feasibility will be measured by (1) number of sites that implement AFFIRM; (2) number of times each site runs the AFFIRM intervention; (3) availability of facilitators; and (4) number of participants that enroll, commence, and complete the intervention. Acceptability will be assessed through mixed-method participant and facilitator evaluations.

#### Secondary

This study’s secondary outcome is implementation fidelity, that is, how closely AFFIRM facilitators adhere to the manualized intervention. This will be assessed through analysis of session audio recordings and facilitator process notes by analysts separate from the Core Facilitation Team.

#### Exploratory

Exploratory outcomes include changes in sexual health self-efficacy and capacity, mental health indicators, internalized homophobia, stress appraisal, proactive and active coping, and hope. These outcomes will be assessed through measures such as: the Sexual Health Capacity Scale [100], the Abstinence and Protection Self-Efficacy Scale [101], the Beck Depression Inventory-II [102], the Diagnostic and Statistical Manual of Mental Disorders-V (DSM-V) Self-Rated Level 1 Cross-Cutting Symptom Measure–Child [103], the Stress Appraisal Measure for Adolescents [104], the Brief COPE [105], the Proactive Coping Inventory for Adolescents-A [106], the Adult Hope Scale [107], the Internalized Homophobia Scale [108], the Current Mood Scale [109], the Everyday Discrimination Scale [110], and the LGBTQ Microaggession Scale [111] (see Table 1).

### Data Collection

AFFIRM participant data are exclusively collected using Web-based surveys, *each of which takes approximately 20 min to complete*. However, data collection takes place in 2 different settings: (1) via tablets (eg, Android tablets) while at the intervention locations (ie, some Web-based screenings, some pre- and posttests); and (2) independently Web-based (eg, some Web-based screenings, some pretests, or follow-ups). Secure collection of data is facilitated by the use of software with secure servers (Qualtrics). Instead of a unique identifier, which participants could forget over the course of the year, participants report their name, age, date of birth, 2 email addresses, gender identity, and sexual orientation at each time point.

### Measures

All measures are completed at all time points, including demographics, as identities and circumstances are generally very flexible for SGMY because of their developmental stage, marginalized sexual and/or gender identities, and contextual circumstances. Measures include the following: (1) *demographics* (eg, age, sexual identity, gender identity, ethno-racial identity, and socioeconomic-status); (2) *sexual health self-efficacy and capacity*, including sexual health capacity [100] and abstinence and protection self-efficacies [101]; (3) *mental health*, including current mood [109], depression [102], and DSM-V cross-cutting symptoms [103]; (4) *coping*, including proactive coping [105] and coping strategies such as active coping, denial, and humor [106]; (5) *stress appraisal*, including perceiving stress as challenge or threat, and seeking out resources to overcome stress [104]; (6) *hope*, including agency and planning to meet goals [107]; (7) experiences with everyday discrimination [110]; (8) *internalized homophobia* [108]; (9) microaggressions, including interpersonal and environmental microaggressions [111]; (10) *AFFIRM Satisfaction Survey,* a 20-item questionnaire developed for AFFIRM completed after intervention delivery, which includes questions regarding (1) satisfaction, (2) overall experience, and (3) suggestions for improvement. For details of adaptation of existing survey measures, please see Table 1.

**Table 1:** Survey instruments.

Construct	Scale, study	Items	Modification
Sexual health self-efficacy and capacity	Sexual Health Capacity Scale [100]	5	Change scale from 5-point (1-5) to 4-point (1-4); Excluded 2 items; Added 1 item: “I understand how my mental and sexual health are connected.”
	Abstinence Self-efficacy Scale [101]	4	Excluded 6 items; Wordings modified
	Protection Self-efficacy Scale [89]	8	Added 4 items: “I can ask for/get a test for HIV and STIs from a doctor, Planned Parenthood, or a clinic.”; “I can read or think about my sexual health plan before having sex.”; “I can access/get information about my sexual health (websites, agencies, trusted adults, professionals, or friends).”; “I can manage my own sexual health.”; Wordings modified
Mental health	Beck Depression Inventory-II [102]	20	No modifications
	DSM-5^a^ Self-Rated Level 1 Cross-Cutting Symptom Measure—Child [103]	20	Excluded 5 items from section 9 (Psychosis) and 10 (Repetitive Thoughts and Behaviors); Slightly modified 1 item
	Current Mode Scale [109]	6	Wordings simplified
Coping	Brief COPE [105]	28	2 items slightly modified
	Proactive Coping Inventory for Adolescents-A—Reflective Coping subscale [106]	11	4 items slightly modified
Stress appraisal	Stress Appraisal Measure for Adolescents [104]	13	1 item excluded; 1 item slightly modified
Hope	Adult Hope Scale [107]	12	No modifications
Internalized homophobia	Internalized homophobia [108]	10	Separated into 2 sections for microaggressions toward sexual orientation and gender identity minorities
Discrimination and microaggressions	Everyday Discrimination Scale [110]	7	2 items excluded; 1 item slightly modified
	LGBTQ^b^ Microaggressions Scale—Interpersonal subscale [111]	10	Excluded 10 items; Separated into 2 sections for microaggressions toward sexual orientation and gender identity minorities
	LGBTQ Microaggressions Scale—Environmental subscale [111]	5	Excluded 2 items; Added 2 items: “In my online environment it was OK to make jokes about LGBTQ+ people.” and “I heard or read someone making fun of chosen pronouns.”

^a^DSM-5: Diagnostic and Statistical Manual of Mental Disorders, 5^th^ edition.

^b^LGBTQ: lesbian, gay, bisexual, transgender, and queer.

### Participant Timeline

Table 2 shows the schedule of events. Participants complete a Web-based screening questionnaire, which includes their preferred site to participate in AFFIRM. For every 2 participants allocated to the intervention-only group, 1 participant is allocated to the wait-list group. The intervention-only group completes 4 data collection time points (pretest, posttest, 6-month follow-up, and 12-month follow-up). The wait-list group completes 5 data collection time points (prewait, pretest, posttest, 6-month follow-up, and 12-month follow-up). The prewait survey is completed immediately following their wait-listed control allocation, so that the pretest for the wait-list group serves as the follow-up on outcomes for the control group. Participants complete the pretests and posttests before the first week and at the last week of the intervention, respectively. The Web-based pretest is completed independently shortly before the first group session. The posttest is completed on tablets (eg, Android tablets) at the intervention location following the final group session. Participants complete the prewait (if applicable), the 6-month follow-up, and the 12-month Web-based independent follow-up. Participants are reminded to participate via email and provided the link to the survey. If participants do not participate following the initial email, up to 2 follow-up emails are sent as reminders for each time point. The follow-up emails are sent at 2 weeks and 4 weeks after the initial email (ie, every 2 weeks for 1 month). If participants indicated in their Web-based screening that SMS text message or phone call is their preferred form of communication, they also receive follow-up SMS text messages at 2 to 4 weeks after the initial email.

**Table 2:** Schedule of events.

Visit details		Screening period			Study period (facilitator meeting; 8 weekly, 1-hour sessions)			Follow-up period	
Visit name		Screening survey	Prewait	Pretest	Orientation	Sessions 1-8	Posttest	6-month follow-up	12-month follow-up
Visit number.		−3	−2	−1	0	1,2,3,4,6,7,8	8	9	10
Week number		-8 to 0	-8 to 0	-8 to 0	0	1-8	8	32	56
Day number		−56 to 0	−56 to 0	−56 to 0	1-56	1-56	1-56	+224 to +238	+392 to +406
Visit window		±56	±14	±0	±0	±0	±0	±14	±14
**Procedures**								
	Written informed consent	X^a^	—^b^	—	—	—	—	—	—
	Entry criteria assessment	X	—	—	—	—	—	—	—
	Participant demographics	X	—	—	—	—	—	—	—
	Group session (intervention or wait-listed control)	X	—	—	—	—	—	—	—
	Facilitator process notes	—	—	—	X	X	—	—	—
	Sexual health self-efficacy and capacity	—	X	X	—	—	X	X	X
	Mental health	—	X	X	—	—	X	X	X
	Internalized queerness	—	X	X	—	—	X	X	X
	Coping	—	X	X	—	—	X	X	X
	Hope	—	X	X	—	—	X	X	X

^a^Procedure conducted.

^b^Not applicable.

### Sample Size

Approximately 300 participants will participate in AFFIRM. Each CCAS has agreed to complete a minimum of 1 AFFIRM iteration, comprising 1 intervention group and 1 wait-list group. This sample was primarily determined to assess the implementation-based outcomes. In addition, this sample would be sufficient for analysis of exploratory objectives, as described below.

### Recruitment

Potential participants are recruited in multiple ways (1) via CCASs and other local community organizations (which are provided with flyers and cards directing potential participants to the independent Web-based screening); (2) via emails to local organizations and community groups serving SGMY (which are provided e-versions of the flyers and cards); and (3) Web-based postings on Facebook and Instagram. The Web-based postings involve geographically and demographically targeted *paid boosts* using Facebook’s Ad Manager.

### Data Management and Monitoring

Data are downloaded from Qualtrics, cleaned, and saved as password-protected files on an encrypted research drive. After data collection and cleaning for the entire study is completed, the data will then be deleted from Qualtrics servers. A data monitoring committee is not required at this stage, as the current phase is primarily focused on implementation acceptability in community sites; however, one will be formed before instigation of a larger trial.

### Harms

As a psychosocial study, risks for adverse effects are negligible or nonexistent. The study’s distress plan, discussed above, will be used if participants present in crisis.

### Ethics and Dissemination

This study has been approved by the University of Toronto’s HIV/AIDS Research Ethics Board (protocol ID#35229). As an uncontrolled, nonrandomized trial, registration at this stage was not completed. If this implementation stage proves promising and the study proceeds to a full-scale multicenter trial, a new protocol will be registered and submitted for publication before participant enrollment.

## Results

It is anticipated that data analyses will use effect size estimates, paired sample *t* tests, and repeated measures linear mixed modeling (LMM) using SPSS to test for differences pre- and postintervention [112-114]. Descriptive analyses will summarize data and profile all variables, including internal consistency estimates. Distributional assumptions, univariate, and multivariate normality of all variables will be assessed. Data determined to be missing at random will be imputed with the expectation-maximization method with importance re-sampling [113].

Clinically significant change estimates and repeated measures LMM will be used to compare the influence of participation in AFFIRM to wait-list on sexual health self-efficacy and capacity, internalized queerness, hope, and depression of SGMY [114]. To test the influence of intervention on multiple outcomes, LMM using both time and interaction terms will be fit using SPSS [115,116]. Repeated measures LMM is considered an improvement over classical repeated measures analyses (eg, repeated measures analysis of variance) because of frequent correlated errors and nonindependence of observations that are forbidden by the assumptions of standard general linear approaches [114-116]. LMM also allows for the exploration of time effects, which is a potential source of confounding because of the partially randomized nature of the design and assumption of no interaction between intervention effects and time [117]. A total of 2 mixed models will be constructed to test the relationship between the intervention condition (AFFIRM group or the treatment as usual or wait-list [TAU/WL] group) and change in outcome variables from pre- to postintervention. Model 1 will include time and condition and will indicate whether there is a significant change in outcome variables over time and if significant cross-sectional associations between treatment condition and outcome variables exist. In model 2, interaction terms will be added to assess the longitudinal associations between treatment condition and the change (slope) of outcome variable scores from pre- to postintervention. The interaction of multiple intersecting identities as well as site, group, and individual-level covariates will be included in this analysis. The impact of behavioral interventions also can be identified through effect size [114]. The potential clinical significance of AFFIRM will be assessed by calculating Cohen *d* effect sizes, comparing percentages of participants at 2 time points. It is expected that compared with the TAU/WL group, the AFFIRM group will show statistically significant change in hypothesized outcomes. The use of similar sample sizes with multiple time points and measures is considered appropriate [112] and allows for analysis of change effects using data points. The qualitative data collected through the acceptability measures will be analyzed using content analysis with ATLAS.ti software by a minimum of 3 coders [118].

## Discussion

AFFIRM is a potentially scalable intervention for SGMY as (1) AFFIRM fosters positive health behaviors by identifying and modifying less healthy behaviors; (2) participants learn how to better cope with minority stressors by rehearsing and having facilitators validate these emerging coping skills; and (3) AFFIRM’s pilot showed positive results on mental and sexual health outcomes [20]. Other CBT-informed group interventions have been effective in reducing adolescent depression and sexual risk, improving mood and behavior, increasing HIV and STI knowledge, and improving self-efficacy [58-60]. This study’s exploratory measures will assess if AFFIRM results in similar outcomes. Group interventions offer SGMY opportunities to learn, observe, and practice skills [79,89], as well as obtain support from peers experiencing similar difficulties [76,77]. Many existing community programs provide safe spaces for SGMY but do not provide skills-based training to deal with the increasingly complex lives of adolescents and young adults This pragmatic trial could make a significant contribution to the field of intervention research by simultaneously moving AFFIRM into practice and evaluating its impact.

## Appendix

Multimedia Appendix 1 Distress protocol for AFFIRM intervention.

Multimedia Appendix 2 AFFIRM crisis response form.

## Abbreviations

CBT: cognitive behavioral therapy
CCAS: collaborating community agency site
DSM: Diagnostic and Statistical Manual of Mental Disorders
LGBTQ: lesbian, gay, bisexual, transgender, and queer
LMM: linear mixed modeling
SGMY: sexual and gender minority youth
STI: sexually transmitted infections
SWWCD: stepped wedge wait-list crossover design
TAU/WL: treatment as usual or wait-list
