# Supplementary material for: An Affirmative Coping Skills Intervention to Improve the Mental and Sexual Health of Sexual and Gender Minority Youth (Project Youth AFFIRM): Protocol for an Implementation Study
Source: JMIR Res Protoc. 2019 Jun 6;8(6):e13462. doi: 10.2196/13462 (PMC6592518; doi:10.2196/13462)
Supplement: Multimedia Appendix 1 [file resprot_v8i6e13462_app1.docx]

# Appendix 1: Distress Protocol for AFFIRM Intervention

***Indications of a Person at Risk (PAR) during AFFIRM group:***

- Indicates experiencing a high level of stress or emotional distress, as detailed in the AFFIRM CRISIS RESPONSE FORM
- Exhibits behaviours suggestive that the group is too stressful (e.g. uncontrolled crying, incoherent speech, indications of flashbacks)
- Indicates they are thinking of hurting themselves/others

***Follow-up actions and questions:***

One group facilitator will:

- Support the participant in distress by leaving the group with them and entering a private office space for further assessment
- Offer time and space - allow participant time to regroup
- Assess mental status using the AFFIRM CRISIS RESPONSE FORM

***Determine if the participant is in imminent danger to self/others (Y or N?)***

1. Based on clinical judgment and using the AFFIRM CRISIS RESPONSE FORM, if a participant’s distress reflects a difficult emotional response that would be reflective of sensitive material/discussions in a group setting, offer support and extend the opportunity to: Stop participation in the group; regroup; or continue with the group. Notify Dr. Craig of actions taken.
2. Based on clinical judgment and using the AFFIRM CRISIS RESPONSE FORM, if a participant’s distress reflects acute emotional distress or safety concerns but is NOT in imminent danger (LOW RISK, MEDIUM RISK), take the following actions: (1) ensure safety using the IMMEDIATE SAFEPLAN; (2) notify emergency contact; (3) suggest participant inform their family physician; (4) document intervention; (4) coordinate follow-up services; and, (5) submit documentation to Dr. Shelley Craig.
3. Based on clinical judgment and using the AFFIRM CRISIS RESPONSE FORM, if a participant’s distress reflects imminent danger (i.e. participant feels unsafe and intends to hurt themselves/others, has an imminent plan and access to means to hurt themselves/others, or HIGH RISK, take the following actions: (1) ensure safety using the IMMEDIATE SAFEPLAN; (2) contact 911/mobile crisis unit, OR co-facilitator will accompany participant to emergency services; (3) notify emergency contact; (4) suggest participant inform their family physician; (5) document intervention; (6) coordinate follow-up services; and, (7) submit documentation to Dr. Shelley Craig.

**Distress Report:**

The group facilitator will submit the AFFIRM CRISIS RESPONSE FORM to Dr. Craig following intervention with the PAR. The AFFIRM CRISIS RESPONSE FORM should be submitted within two days of the intervention with the PAR. The more detailed the report the better. The group facilitator should focus on including as much detail on what the participant said as possible, using their own words. All reports need to be stored in a secure and password protected location.

**Withdrawal from the Study:**

Based on clinical judgment and using the AFFIRM CRISIS RESPONSE FORM, if a participant’s distress is deemed as grounds for withdrawal from the study (i.e., in crisis and imminent danger to self/others), the group facilitator will explain to the participant that they will be withdrawn from the study once the participant is stabilized.

The following SCRIPT will be used with participants to explain withdrawal from the study:

“How are things going? Do you feel you have the supports you need right now? As a participant in the AFFIRM study, we want to make sure you are supported as best as possible. AFFIRM is about providing support to LGBTQ+ youth. Unfortunately, we will have to withdraw you from the study to make sure you get the best possible support at this time. [If currently participating in the AFFIRM intervention]: Do you feel you are able to continue participating in the group for the remaining weeks? You are always welcome to access the AFFIRM manual and resources online at (provide website address on a card). Do you have any questions about being withdrawn from the study? Is there anything else we can do?”

*Adapted from Draucker, C. B., Martsolf, D. S., & Poole, C. (2009). Developing distress protocols for research on sensitive topics. Archives of psychiatric nursing, 23(5), 343-350.*
