# Supplementary material for: An Affirmative Coping Skills Intervention to Improve the Mental and Sexual Health of Sexual and Gender Minority Youth (Project Youth AFFIRM): Protocol for an Implementation Study
Source: JMIR Res Protoc. 2019 Jun 6;8(6):e13462. doi: 10.2196/13462 (PMC6592518; doi:10.2196/13462)
Supplement: Multimedia Appendix 2 [file resprot_v8i6e13462_app2.docx]

# Appendix 2: AFFIRM Crisis Response Form

| **AFFIRM CRISIS RESPONSE FORM** | | | | | | | |
| --- | --- | --- | --- | --- | --- | --- | --- |
| PARTICIPANT NAME: | | | | | AGE: | | |
| PARTICIPANT PHONE NUMBER: | | **FOLLOW-UP DATE:** | | | | | |
| GROUP SITE: | | | | | INCIDENT DATE: | | |
| STAFF NAME: | | | | | REPORT DATE: | | |
| **WARNING SIGNS DISPLAYED** | | | | | | | |
| - Change in attendance | - Inability to concentrate | | | | - Inability to complete assignments | | |
| - Lack of interest/ withdrawal | - Change in relationships with classmates | | | | - Increase in irritability or aggressiveness | | |
| - Wide mood swings | - Unexpected displays of emotions | | | | - Despairing attitude | | |
| - Preoccupation with death and suicide (writing about it, drawing images of death) | - Sleep disturbances, loss of appetite | | | | - Changes in appearance and personal care | | |
| - Other: | | | | | | | |
| **CURRENT FACTORS- what will keep you safe RIGHT NOW?** | | | | | | | |
| SETTING THE STAGE:   1. Be clear about the purpose of the safety plan, and state limits of confidentiality 2. Be calm and non-judgmental | | | | | | | |
| ASK:  “Are you thinking of killing yourself”   1. If “No”: “My reason for asking is _______________. If you were to start feeling like you would potentially hurt yourself, what would you do to help yourself?” Continue with HLP (History, Loss, Plan). 2. If “Yes” or “Maybe” continue with HLP (History, Loss, Plan), and CURRENT PLAN | | | | | | | |
| **HLP- HISTORY, LOSS, PLAN**  HISTORY/PERVIOUS ATTEMPTS   - Have you felt like this before? - How close have you come to killing yourself? What happened? - Do you know anyone who has killed themselves?   LOSS/ALONENESS   - Do you feel a sense of loss? - Who do you feel you can really talk to? - Is there anything in your life that makes you want to live (pets, significant people, goals, etc.)? - Do you belong to any organizations or groups in the community?   PLAN   - Do you have a plan to kill yourself? - If YES, continue with CURRENT PLAN - If NO, continue to IMMEDIATE SAFEPLAN | | | | | | | |
| **CURRENT PLAN**  *If the Person at Risk (PAR) is NOT able to participate in the intervention, activate emergency response and 24-hrmonitoring services* | | | | | | | |
| Do you have a current plan to kill yourself? Or harm someone else? | | YES | | | | NO | |
| If YES, what is your plan? | | | | | | | |
| What things have you done to get ready? | | | | | | | |
| How soon? | | | | | | | |
| What life/situational factors are contributing to these feelings now? | | | | | | | |
| Are you experiencing physical and emotional pain? | | | | | | | |
| If the PAR has a plan, work with them to disable the plan.  List interventions to disable plan here: (who, what, when, and how) | | | | | | | |
| **ASSESS** | | | | | | | |
| LOW RISK   - Suicide ideation - Does not have clear plans/means - Feels hopeless and/or helpless, but has support in place | MEDIUM RISK   - Indicated suicide intent - May have plan/means - Perceived support - Future investment | | | HIGH RISK   - Suicide intent - Has plans/means/time frame - No perceived supports - Perception of loss isolation/aloneness | | | |
| **ACT** | | | | | | | |
| LOW RISK RESPONSE   - Ensure safety - Notify emergency contact - Document intervention - Coordinate follow-up services - Submit documentation to Dr. Shelley | MEDIUM RISK RESPONSE   - Ensure safety - Notify emergency contact - Suggest the participant contact family physician - Document intervention - Coordinate follow-up services - Submit documentation to Dr. Shelley | | | HIGH RISK RESPONSE   - Ensure safety - Contact 911/mobile crisis unit, OR - Co-facilitator will accompany to emergency services - Notify emergency contact - Document intervention - Coordinate follow-up services - Submit documentation to Dr. Shelley | | | |
| **IMMEDIATE SAFEPLAN** | | | | | | | |
| Are you currently engaged in any mental health treatment or other counselling support? | | | | YES | | | NO |
| Have you had any pervious mental health or concerns where you sought counselling? | | | | YES | | | NO |
| What have you learned from current or past counselling or mental health supports that might help with keeping safe now? If PAR agrees connect/contact current or past counsellor for additional ongoing supports  List here: | | | | | | | |
| **What is doable now?**  Situational changes that disable the plan and the difficult situation:        What personal strengths are available to the PAR now? | | | | | | | |
| Establish safety contacts | YES | | | NO | | | |
| Who is able, available and acceptable? (list name and contact info) | | | | | | | |
| Emergency Safety Contact (List medical doctor and local crisis line numbers) | | | | | | | |
| Crisis Response Form submitted to Dr. Shelley Craig | | | Date report received: | | | | |
| Received and reviewed by Dr. Shelley Craig | | | Signature: | | | | |

*Adapted from Chilliwack School District, Ministry for Child and Family Development: Child & Youth Mental Health, Fraser Health Authority: Adolescent Crisis Response Program, and The F.O.R.C.E. (2011). Community Suicide Prevention Protocol for Children & Youth, 28-30. Retrieved from childandyouth.com.*

*Adapted from A Collaborative Response for Children In Crisis (2015). Community SPIRR Protocol: Suicide Prevention, Intervention, and Risk Review Protocol, Appendix A: Student Suicide Safety Review. Retrieved from cdsbeo.on.ca.*
